# Profile of the *tprK* gene in primary syphilis patients based on next-generation sequencing

**DOI:** 10.1371/journal.pntd.0006855

**Published:** 2019-02-21

**Authors:** Dan Liu, Man-Li Tong, Xi Luo, Li-Li Liu, Li-Rong Lin, Hui-Lin Zhang, Yong Lin, Jian-Jun Niu, Tian-Ci Yang

**Affiliations:** 1 Center of Clinical Laboratory, Zhongshan Hospital, School of Medicine, Xiamen University, Xiamen, China; 2 Institute of Infectious Disease, School of Medicine, Xiamen University, Xiamen, China; 3 Zhongshan Hospital, Fujian Medical University, Xiamen, China; Institut Pasteur, FRANCE

## Abstract

**Background:**

The highly variable *tprK* gene of *Treponema pallidum* has been acknowledged to be one of the mechanisms that causes persistent infection. Previous studies have mainly focused on the heterogeneity in *tprK* in propagated strains using a clone-based Sanger approach. Few studies have investigated *tprK* directly from clinical samples using deep sequencing.

**Methods/Principal findings:**

We conducted a comprehensive analysis of 14 primary syphilis clinical isolates of *T*. *pallidum* via next-generation sequencing to gain better insight into the profile of *tprK* in primary syphilis patients. Our results showed that there was a mixture of distinct sequences within each V region of *tprK*. Except for the predominant sequence for each V region as previously reported using the clone-based Sanger approach, there were many minor variants of all strains that were mainly observed at a frequency of 1–5%. Interestingly, the identified distinct sequences within the regions were variable in length and differed by only 3 bp or multiples of 3 bp. In addition, amino acid sequence consistency within each V region was found among the 14 strains. Among the regions, the sequence IASDGGAIKH in V1 and the sequence DVGHKKENAANVNGTVGA in V4 showed a high stability of inter-strain redundancy.

**Conclusions:**

The seven V regions of the *tprK* gene in primary syphilis infection demonstrated high diversity; they generally contained a high proportion sequence and numerous low-frequency minor variants, most of which are far below the detection limit of Sanger sequencing. The rampant variation in each V region was regulated by a strict gene conversion mechanism that maintained the length difference to 3 bp or multiples of 3 bp. The highly stable sequence of inter-strain redundancy may indicate that the sequences play a critical role in *T*. *pallidum* virulence. These highly stable peptides are also likely to be potential targets for vaccine development.

## Author summary

Variations in *tprK* have been acknowledged to be the major contributors to persistent *Treponema pallidum* infections. Previous studies were based on the clone-based Sanger approach, and most of them were performed in propagated strains using rabbits, which could not reflect the actual heterogeneous characteristics of *tprK* in the context of human infection. In the present study, we employed next-generation sequencing (NGS) to explore the profile of *tprK* directly from 14 patients with primary syphilis. Our results showed a mixture of distinct sequences within each V region of *tprK* in these clinical samples. First, the length of identified distinct sequences within the region was variable, which differed by only 3 bp or multiples of 3 bp. Then, among the mixtures, a predominant sequence was usually observed for each V region, and the remaining minor variants were mainly observed at a frequency of 1–5%. In addition, there was a scenario of amino acid sequence consistency within the regions among the 14 primary syphilis strains. The identification of the profile of *tprK* in the context of human primary syphilis infection contributes to further exploration of the pathogenesis of syphilis.

## Introduction

Syphilis, caused by *Treponema pallidum* subsp. *pallidum*, is an ancient sexually transmitted disease that was initially recognized in the 15^th^ century and is a public health threat that cannot be neglected [1, 2]. The completion of the first whole-genome sequencing of the Nichols strain of *T*. *pallidum* provided a wealth of information about the characteristics of this pathogen [3], since then the sequence of other laboratory treponemal strains has also been released [4–12]. These particular achievements have revealed slight variations among different strains in a small genome (∼1.1 Mb), and most of the genetic diversity occurs in six genomic regions, including a polymorphic multigene family encoding 12 paralogous proteins (*tpr A* through *tprL*), highlighting most likely a factor in the pathogenesis of *T*. *pallidum* [2, 6, 13].

Within the *tpr* family, the antigen-coding *tprK* has been found to be the direct target of the human immune response [14], although its surface exposure has been challenged and remains to be fully confirmed [15–17]. Several remarkable studies performed in the rabbit model have demonstrated that the *tprK* gene possesses high genetic diversity at both the intra- and inter-strain levels, and the genetic variation in *tprK* is localized to seven variable regions (V1-V7) flanked by highly conserved domains [18–20]. Theoretically, through gene conversion, variations in the V regions would generate millions of chimeric *tprK* variants, resulting in a constant alteration in the *T*. *pallidum* antigenic profile [21]. Therefore, the *tprK* gene is acknowledged to have a pivotal role in immune evasion and pathogen persistence [22, 23].

Previous studies focusing on the genetic variability of *tprK* were mainly based on the clone-based Sanger approach; when using this approach, it would inevitably encounter a bottleneck in clone selection where minor variants, especially at low frequencies, are lost; consequently, the complete mutation profile of *tprK* is not fully understood. Furthermore, except for one recent publication that reported on whole-genome sequencing directly from clinical samples of *T*. *pallidum* to investigate how *tprK* diversifies in the context of human infection [24], other *tprK-*related studies were conducted based on rabbit-derived samples [18, 19, 25, 26].

In the present study, we seek to systematically reveal the profile of *tprK* in *T*. *pallidum* directly from patients with primary syphilis by employing next-generation sequencing (NGS), thus providing important insights into the understanding of the diversity of *tprK* directly from primary syphilis patients and contributing to further explorations of the mechanisms of long-term *T*. *pallidum* infection.

## Methods

### Ethics statement

All participants in this study were adults and written consent was obtained with signatures from all patients in accordance with institutional guidelines prior to the study. The study was approved by the Ethics Committee of Zhongshan Hospital, Xiamen University, after a formal hearing and was in conformance with the Declaration of Helsinki.

### Sample collection

Swab samples were obtained from the skin lesions of 14 patients (X-1~14) with primary syphilis. The clinical diagnosis of syphilis was based on the US Centers for Disease Control and Prevention (CDC) [27] and the European CDC (ECDC) guidelines [28].

### Isolation of DNA

DNA was extracted from the swab samples using the QIAamp DNA Mini Kit (Qiagen, Inc., Valencia, CA, USA) according to the manufacturer’s instructions, and careful precautions were implemented to avoid DNA cross-contamination between isolates [11]. Each sample was quantified by targeting *tp0574* through qPCR using a 96-well reaction plate with a ViiA 7 Real-Time PCR System (Applied Biosystems, USA). For the absolute quantification of treponemal copies, a standard curve was constructed using 10-fold serial dilutions of cloned plasmids (for *tp0574*) generated through TOPO TA technology (Invitrogen, Carlsbad, CA, USA) and transformation of DH5α *Escherichia coli* cells [29]. The DNA samples that tested positive were used to amplify *tp0136* to determine whether these 14 clinical stains belong to the Nichols-like group or SS14-like group [30].

### Segmented amplification of the *tprK* gene

First, the extracted DNA was directly used in the amplification of the *tprK* full open reading frame (ORF). The primers used for the amplification are listed in S1 Table. For amplification, KOD FX Neo polymerase (Toyobo, Osaka, Japan) was used. The reaction mixture contained 25 μL of 2× PCR buffer, 0.4 mM deoxynucleotide triphosphates, 0.3 μM of each primer, 1 U of KOD FX Neo polymerase, and 5 μL of genomic DNA in a final volume of 50 μL. The cycling conditions were as follows: 94°C for 2 min, followed by 40 cycles of 98°C for 10 s, 60°C for 30 s, and 68°C for 30 s. Then, the amplicons were gel purified and stored at -20°C for further processing as the template for segmented amplification described below.

Second, partial amplification of four fragments of 400–500 bp, overlapping by at least 20 bp, covered *tprK* ORF. The primers are listed in S1 Table. The purified full length *tprK* amplicons were diluted 1000-fold and used as a segmented amplification template. The amplification mixture was the same as described above except that the primers were 0.15 μM. The cycling conditions were denaturation at 94°C for 2 min, followed by 30 cycles of 98°C for 10 s, 55°C for 30 s, and 68°C for 30 s. The size of all the products was verified by 2% agarose gel electrophoresis, and the products were gel purified. The four subfragments corresponding to each sample were mixed in equimolar amounts into one pool to produce a separate library using a barcode to distinguish each sample.

### Library construction and next-generation sequencing

Library construction and sequencing were performed by the Sangon Biotech Company (Shanghai, China) on the MiSeq platform (Illumina, San Diego, CA, USA) in paired-end bi-directional sequencing (2×300 bp) mode. FastQC (http://www.bioinformatics.babraham.ac.uk/project/fatsqc/) and FASTX (http://hannonlab.cshl.edy/fastx_toolkit) tools were applied to check and improve the quality of the raw sequence data, respectively. The final reads collected from 14 patients were compared with the *tprK* of the Seattle Nichols strain (GenBank accession number AF194369.1) using Bowtie 2 (version 2.1.0).

Based on the previously published principle that was used to extract sequence [24], an in-house Perl script was developed and applied to specifically capture DNA sequences within seven regions of the *tprK* gene across 14 strains from raw data, both forward and reverse. Briefly, the user-defined strings that matched the conserved sequence flanking the variable regions were used to catch the variable sequences. The defined strings referred to the mapping result of the reference and should be as long as necessary to ensure specificity (approximately 12–16 bp). Thus, the exact number of distinct sequences within seven regions of the *tprK* gene from each sample was acquired. The intrastrain heterogeneous sequences were valid if the following conditions were simultaneously verified for any variant sequence: 1) being supported by at least fifty reads and 2) displaying a frequency above 1%. Then, the relative frequency of the sequences within each variable region was calculated.

### Accession numbers

The raw data sequences of these 14 primary syphilis samples were deposited in the SRA database (BioProject ID: PRJNA498982) under following BioSample accession numbers: SAMN10340238- SAMN10340251 for X-1-X-14, respectively.

## Results

### Description of clinical samples and *tprK* sequencing by NGS

The samples (N = 14) were collected from patients diagnosed with primary syphilis at Zhongshan Hospital, Xiamen University. The clinical data of patients are shown in Table 1. The qPCR data of *tp0574* showed that the number of treponemal copies in each clinical sample was eligible for the amplification of the *tprK* full ORF. And based on the sequencing data of *tp0136*, most of them belonged to SS14-like group and only two belonged to the Nichols-like group. The median sequencing depth of the *tprK* segment samples ranged from 10568.99 to 56676.38, and the coverage ranged from 99.34% to 99.61%, showing high identity with the *tprK* gene of the Seattle Nichols strain.

**Table 1:** Description of clinical samples and *tprK* sequencing by NGS.

Isolate	Gender	Age (year)	Serum RPR titer	Serum TPPA	Dark field microscopy	*T*. *pallidum* genome copies by *tp0574*	Genetic group by *tp0136*	Total reads	On-target reads (%)	Mean depth of coverage
X-1	Male	45	1:16	+	Positive	8.2E+03	SS14-like group	357382	99.41	51967.28
X-2	Male	27	1:16	+	Positive	8.82E+04	Nichols-like group	340240	99.47	49660.18
X-3	Male	62	1:16	+	Positive	4.55E+04	SS14-like group	398898	99.41	56676.38
X-4	Male	65	1:4	+	Positive	1.15E+04	SS14-like group	365060	99.34	52742.09
X-5	Male	76	1:16	+	Positive	5.73E+04	SS14-like group	363940	99.61	52960.83
X-6	Male	64	1:32	+	Positive	2.33E+02	SS14-like group	106934	99.37	14249.15
X-7	Female	56	1:16	+	Positive	1.26E+04	Nichols-like group	114012	99.37	15579.12
X-8	Male	46	1:4	+	Positive	1.41E+04	SS14-like group	103280	99.43	12951.11
X-9	Male	40	1:4	+	Positive	1.39E+03	SS14-like group	119552	99.43	15864.28
X-10	Male	66	1:32	+	Positive	9.17E+03	SS14-like group	114064	99.37	14927.08
X-11	Male	44	1:2	+	Positive	2.67E+02	SS14-like group	94572	99.50	12935.89
X-12	Male	39	-	+	Positive	6.40E+03	SS14-like group	114588	99.43	14944.66
X-13	Male	63	1:16	+	Positive	2.02E+02	SS14-like group	118634	99.37	15013.54
X-14	Male	61	1:1	+	Positive	1.16E+03	SS14-like group	82812	99.37	10568.99

Abbreviations: NGS, next-generation sequencing; RPR, reactive plasma reagin; TPPA, *T*. *pallidum* particle agglutination; +, positive;-, negative.

### Sequence variability of *tprK* directly from primary syphilis samples

#### The number and length variation of distinct sequences in seven regions of the *tprK* gene

**Fig 1:**
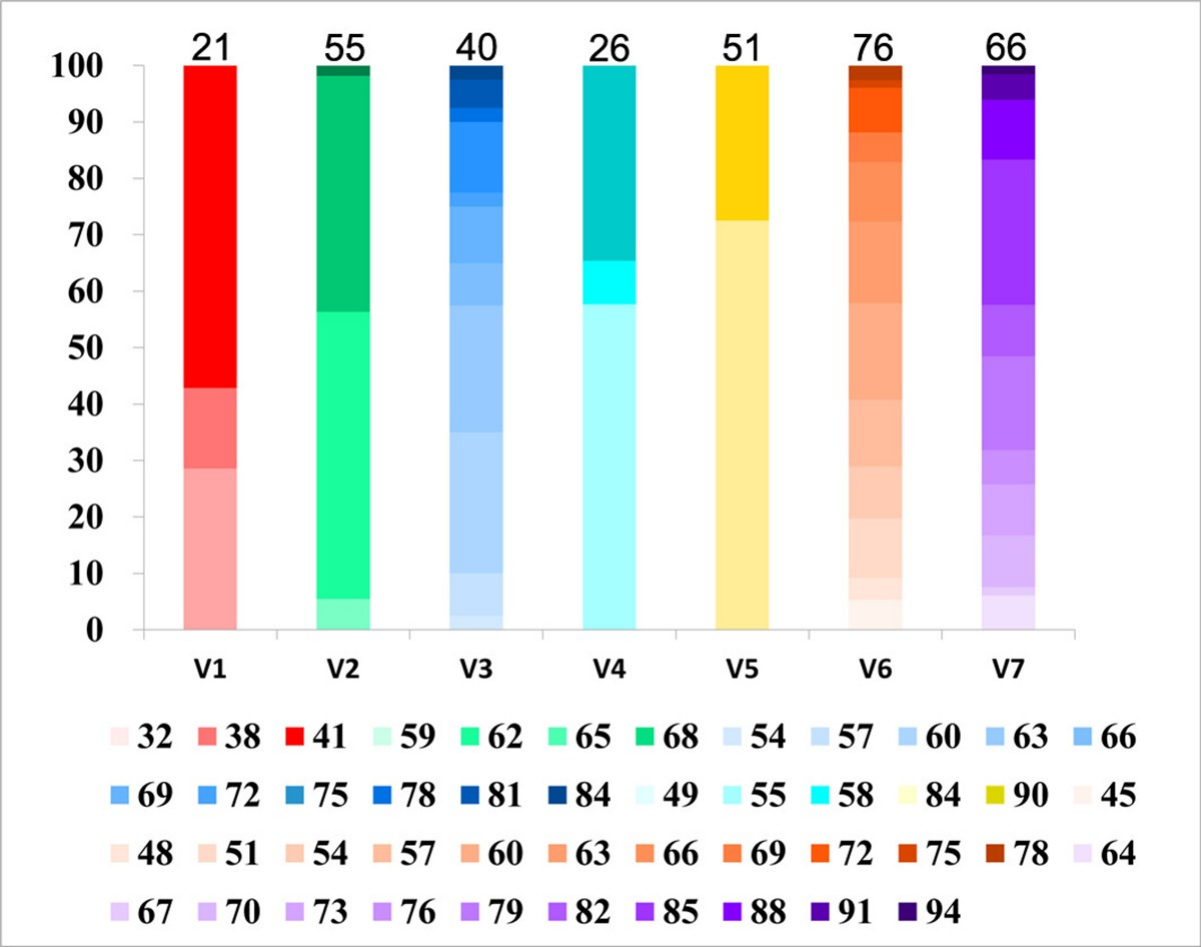
The varied length forms within each V region of *tprK* across all the samples. The varied length forms within each V region are presented as the frequencies in each region and are filled with the gradient colour. Each colour indicates a length form involving several polymorphic sequences. The sum of different nucleotide sequences captured in each V region within each sample is also shown above the V region.

According to the strategy, we extracted sequences from seven V regions to evaluate the sequence variability of *tprK* directly from primary syphilis samples. Different nucleotide sequences within each V region from each sample were all included in the analysis, and up to a total of 335 nucleotide sequences were captured. The number of distinct sequences in the seven regions of the *tprK* gene ranged from 21–76, with the highest number in V6 and the lowest in V1 across all samples (Fig 1 and S2 Table). The length of the captured sequences within each V region was also found to be variable, particularly in V3, V6 and V7, with 11 or 12 forms. In contrast, the length of the sequence in V5 had only two forms, namely, 84 bp and 90 bp. When the length of all sequences within each sample was calculated, the length of all differed by 3 bp or multiples of 3 bp. Interestingly, although the lengths of V3, V6 and V7 were particularly variable across all populations, these lengths continued to change by 3 bp. In this regard, the lengths of V1, V4 and V5 appeared to vary in intervals of 6 bp.

#### The proportion distribution of distinct sequences in seven regions of the *tprK* gene

**Fig 2:**
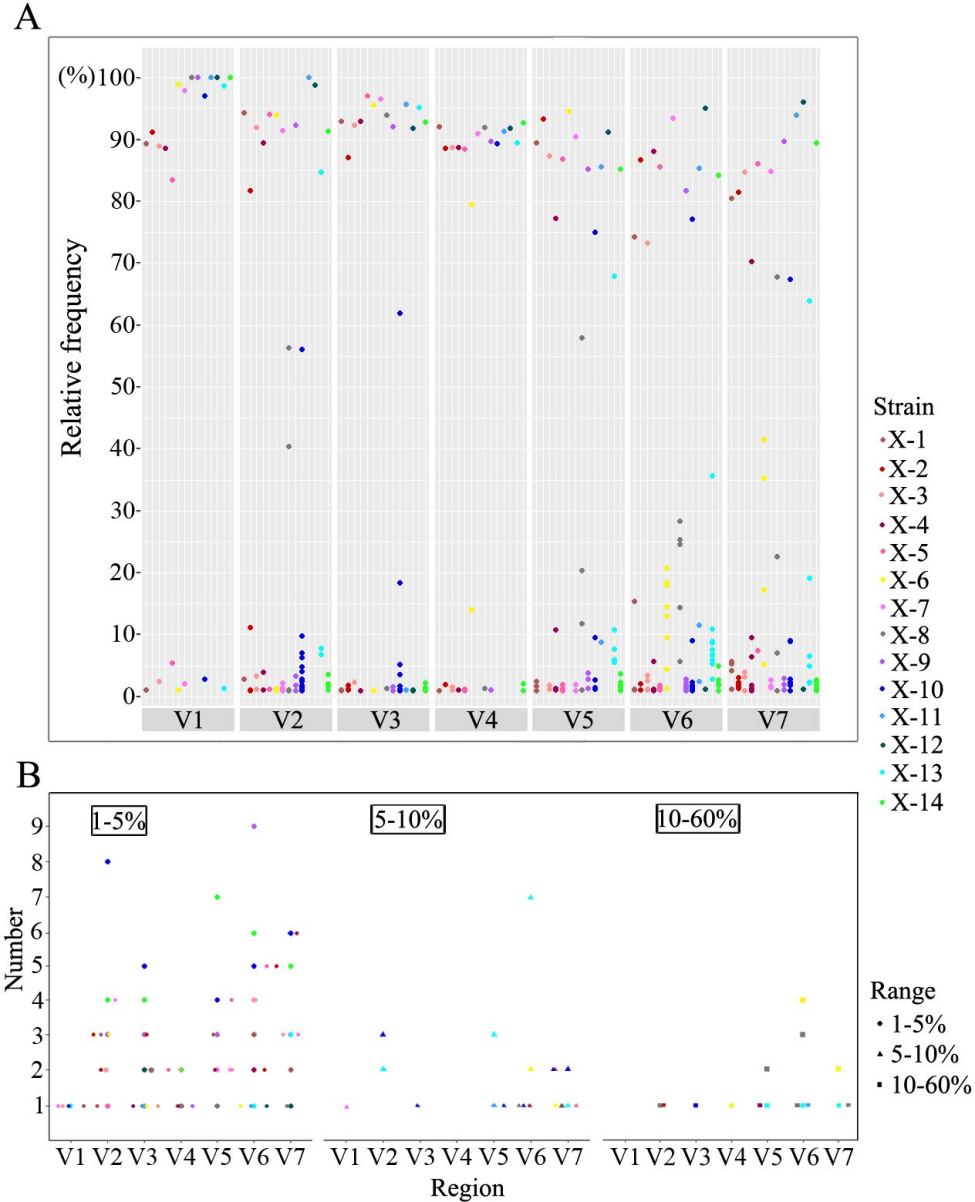
The proportion distribution of distinct sequences within each V region of *tprK* from each sample. (A) The dots indicate the relative frequency of identified distinct sequences within each V region of *tprK* from each clinical sample, and the colour specifies the strain. (B) The graph shows the number of minor variants within each V region. The three thresholds (1–5%, 5–10% and 10–60%) are characterized by three different shapes, and the colour specifies the strain.

The captured sequences were ranked by relative frequency within each V region of each strain. As Fig 2A shows, there was a predominant sequence in each V region of ten samples directly from primary syphilis patients, and the proportion of this sequence was almost above 80%. While the frequency of the predominant sequence in some V regions of four samples (X-6, 8, 10, 13) was lower than 60%, and the frequency ranged from 20–60%. Then the frequency was found to be decreased in the V2, V5, V6 and V7 regions, and the frequency in V6 of X-6 was even lower at 20.8%.

Apart from the detected predominant sequence within seven V regions, there was still a mixture of minor variants in each V region. Altogether, the frequency of all detected minor variants was almost below 20% (231/237) (Fig 2A). To investigate the exact relative frequency distribution of minor variants, we used three thresholds to explore the characteristics (Fig 2B). The major proportion of the variants in primary syphilis samples was in the 1–5% (181/237) range, and the lowest was in the 10–60% (22/237) range. At the two thresholds (5–10% and 10–60%), the observed variants were all mainly in V2, V5, V6 and V7 and from 4 samples (X-6, 8, 10, 13). This observation corresponded to the lower proportion of their predominant sequences.

### Inter-population redundancy of the deducted amino acid sequence

**Fig 3:**
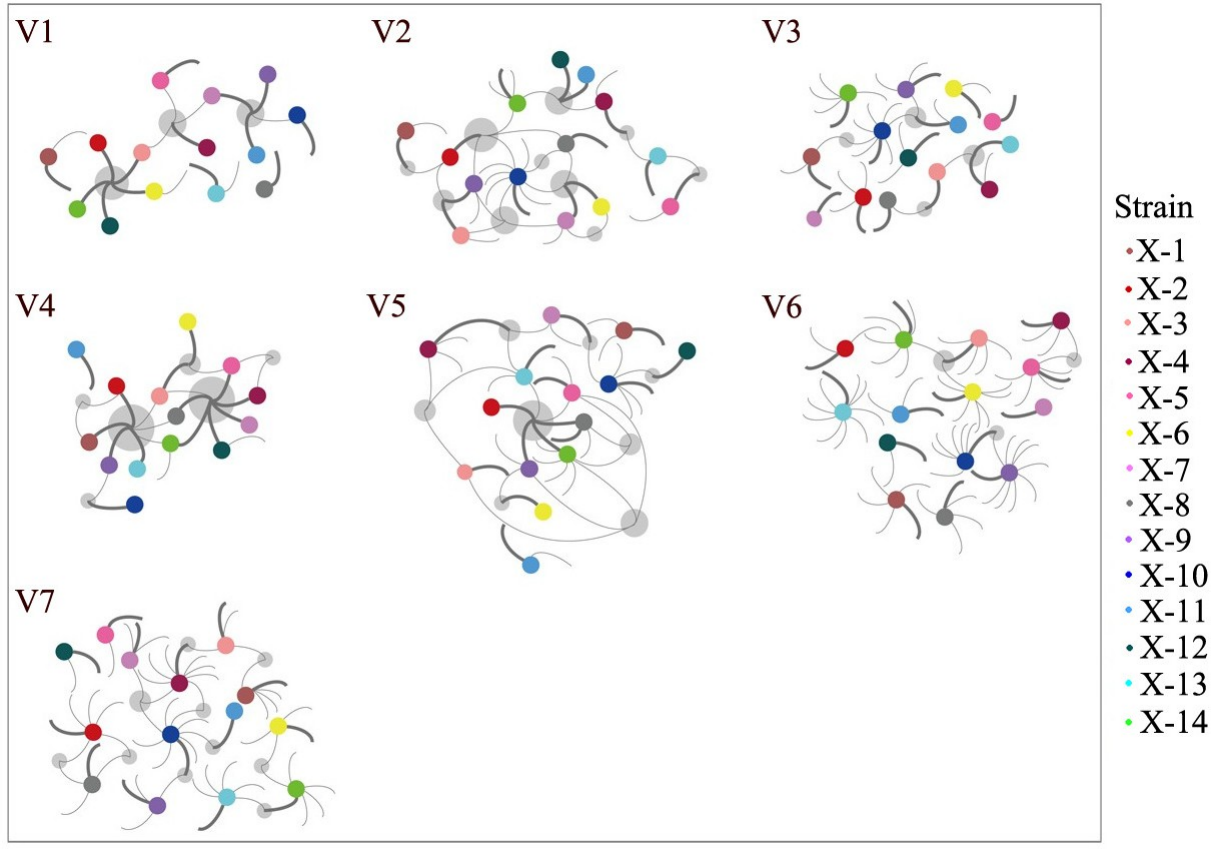
The scenario of redundant *tprK* amino acid sequences among all 14 primary syphilis clinical samples. The 14 strains are specified by coloured solid circles, and the predominant sequence and minor variants within each V region of one strain are represented by a bold arc and thin arcs, respectively. Each grey circle indicates the occurrence of sequence consistency among the strains.

Nucleotide sequences found in variable regions were translated into amino acid sequences *in silico*. In eight cases, two or more amino acid sequences were found to be identical in one sample although they were coded by different nucleotide sequences (S3 and S4 Tables). No sequence yielded a *tprK* frame shift or premature termination. When distinct sequences within each V region from each strain were compared, a scenario of sequence consistency was found. As Fig 3 shows, V1 and V4 presented a strong shared sequence capacity. The sequence IASDGGAIKH in V1 was observed in five strains (5/14) and DVGHKKENAANVNGTVGA in V4 was shared across seven strains (7/14). However, the parallel sequences in V3 and V6 did not seem as significant as in other V regions, especially in V6.

To further explore whether the shared scenario was usually displayed by the predominant sequence across all the strains, we involved only the predominant sequence in the V region of each sample, which was represented by the bold arc in Fig 3 and found that V1 and V4 still presented similar shared sequence abilities despite the decreased redundant sequences. The occurrence of the consistent sequence in V1 and V4 could reach five strains and six strains, respectively (Table 2). For the V3 and V6 regions, which were rarely consistent with sequences, the shared sequence in V3 occurred only between two strains, and there was no consistent sequence found in V6. Meanwhile, there was also no redundant sequence observed in V7.

**Table 2:** The shared predominant amino acid sequences in V1 and V4 of *tprK* among 14 primary syphilis samples.

Region	The amino acid sequence	Strain	Frequency (%)
V1	IASDGGAIKH	X-2	91.2
		X-3	89
		X-6	98.9
		X-12	100
		X-14	100
	IASEDGSAGNLKH	X-7	97.9
		X-9	100
		X-11	100
			
V4	DVGHKKENAANVNGTVGA	X-4	88.7
		X-5	88.5
		X-7	90.9
		X-8	92
		X-12	91.8
		X-14	92.7
	DVGRKKDGAQGTVGA	X-1	92.1
		X-2	88.5
		X-9	89.7
		X-13	89.5
	DVGHKKDGAQGTVGA	X-3	88.7
		X-6	80.0

## Discussion

Although a recent landmark study has reported the successful long-term *in vitro* propagation of *T*. *pallidum* [31], research on this pathogen has been greatly hindered by the lack a system for genetic manipulations in past decades [19, 32]. The whole genome sequencing of the Nichols strain of *T*. *pallidum* provided a new perspective for the study of treponemal genes and proteins. Among these genes, *tprK* has been extensively studied because of its highly variable antigenic profile. In the present study, we performed NGS, a more sensitive and reliable approach, to gain better insight into the profile of *tprK* in primary syphilis patients. Overall, there was a sequence mixture concentrated on seven variable regions of *tprK* in primary syphilis samples. Among the seven V regions, V1 and V6 were found to have the lowest and highest variability, respectively (Figs 1 and 2A), which was consistent with the findings of previous studies [24, 33]. Although *tprK* was previously revealed to have rampant genetic diversity within each strain, the exact proportion of these variant sequences within one strain would not be clearly known by using previous clone-based Sanger approach [18, 19, 25]. In fact, we also applied the clone-based Sanger approach to analyse the *tprK* gene in this research. As described in Pinto *et al*.’ study [24], it generally displayed the predominant sequence within each V region (consistent with the sequence found by NGS) but could not identify all the minor variants (S1 Fig). However, it is an advantage of NGS to fully discover the variants [34, 35]. Combined with the use of an in-house Perl script, we were able to retrieve the variants within the regions of each strain and calculate the relative frequency of the variants, thus disclosing the proportion of these variant sequences in primary syphilis patients.

As shown in Fig 2, the distribution of variants within the V regions of *tprK* from primary syphilis patients reveals that the vast majority of them have a high proportion of predominant sequences (frequency above 80%) and numerous minor variants (frequency below 20%), but very few sequences have a frequency between 20% and 80%. Moreover, these minor variants were found to be mostly distributed at a frequency of 1–5% (Fig 2B), which was extremely below the detection limit for Sanger sequencing [36]. This feature may represent a logical fitness-based evolution where high-frequency sequences are better fitted to avoid immune recognition and numerous low-frequency minor variants may simply emerge and most of them would likely disappear if they were not advantageous for syphilis developing [37]. It is worth noting that the sequences appearing between the frequency of 20–80% were mainly concentrated in the V2, V5, V6 and V7 regions mostly from X-6, 8, 10, 13 (Fig 2). The distribution pattern of these variants from these samples may suggest that with disease progression or increasing immunity, the balance of the original sequence distribution was broken and some V regions (e.g., V2, V5, V6 and V7) began to change. As a result, a minor variant (or a new variant) became advantageous and its frequency gradually increased, ultimately replacing the original predominant sequence, which further promoted the antigenic diversity of TprK for *T*. *pallidum* to escape immune clearance and potentially leading to the development of late syphilis, neurosyphilis or serofast status [15, 21, 38, 39]. Additionally, among these four V regions, the frequency of the predominant sequence in V6 was particularly low (Fig 2A), suggesting that V6 may be the first affected region and is involved in immune evasion during the course of infection [21, 24].

In this study, besides the distinct variations in *tprK* sequences, we also found length heterogeneity in this gene (Fig 1). The size range of the captured sequences was the largest for V3, V6 and V7, which was similar to the findings of Pinto *et al*. [24], demonstrating that the variations in these three regions could more easily cause changes in length. Nevertheless, the diversity of length forms was much lower than the diversity of variants within each V region. Especially in the V5 region, there were many different variants observed, but only two lengths (84 and 90bp) were present, which was also observed in the previous study [21]. Additionally, it was interesting that the length of all distinct sequences differed by only 3 bp or multiples of 3 bp, and previous research data also supported this pattern change [21, 24]. A change pattern characterized multiple of 3 bp matched the triplet codon in protein coding, which has made us think that this feature probably explains why it is rare to uncover a *tprK* frame shift. In fact, no frameshifts were detected in our research and only one was detected in the study of Pinto *et al*. [24]. Additionally, synonymous nucleotide sequence of *tprK* was rare and was found only in the V2 and V5 regions (S3 and S4 Tables), in accordance with the study by Pinto *et al*. [24]. These phenomena suggest that the rampant diversity of *tprK* could be regulated by a strict gene conversion mechanism to avoid yielding an abnormal detrimental antigen for *T*. *pallidum*.

A dominant amino acid sequence for a specific V region in one patient depends on the immune response of that specific patient. For this reason, it may be difficult to find out several syphilitic patients for which the amino acid sequences for some V regions are exactly the same. Actually, despite the significant polymorphic characteristic of *tprK*, at least half of the strains had sequences shared by other strains (Fig 3) in our study, which was similar to previous findings [24]. And *tprK* inter-population redundancy was maintained at a high level in V1 and V4 in contrast to other regions, especially when only the predominant sequence within each V region was analysed (Table 2). Interestingly, the most stable amino acid sequence (IASDGGAIKH) of inter-population redundancy in V1 among 14 primary syphilis patients was also found to be the most frequent sequence in the 24 syphilis patients in Pinto’s study [24]. And the sequence (DVGHKKENAANVNGTVGA) in V4 was also found at a moderate proportion in share among the 24 clinical samples. The similar findings that were observed between the two studies using different approaches to investigate the adaptive traits of the pathogen during different human infection were exciting and clearly suggest the existence of better fitted antigenic profiles to address the immune response of the host. In previous studies [15, 38, 40], the molecular localization in the N-terminal region of *tprK* was conformed to displayed promising partial protection in a rabbit model. Therefore, the highly stable shared peptide of V1 and V4 across all the strains would likely be a potential target for vaccine development.

Finally, the limitations of our research should be discussed. First, the findings reported above were based on amplicons of *tprK*. The possible introduction of errors by polymerases used for the amplification of templates for NGS could not be ignored, although the data showed that the error was minimal. Second, this study provides information on individual V regions instead of information on a single *tprK* ORF. It would not be correct to assume that certain nucleotide sequences within the V regions are derived from a same single *tprK* ORF, as this would result in artificial sequences.

In summary, the characteristic profile of *tprK* in primary syphilis patients was unveiled to generally contain a high proportion sequence and many low-frequency minor variants within each V region. The variations in V regions were regulated by a strict gene conversion mechanism to keep the length differences to 3 bp or multiples of 3 bp. The findings could provide important information for further exploration of the role of *tprK* in immune evasion and persistent infection with syphilis. Furthermore, the peptides in each V region, especially the highly conserved peptides found in this study, could serve as a database of B cell epitopes of TprK for human immunological studies in the future.

## Supporting information

S1 Fig Comparison of the results of NGS and clone-based Sanger sequencing in V6 of the X-8 strain. RF values indicate the relative frequency of each sequence.
(TIF)

S1 Table The primers for *tprK* amplification. (DOCX)

S2 Table A sum of the lengths of distinct nucleotide sequences within each V region of *tprK* from each sample. (XLSX)

S3 Table The nucleotide sequences within the seven variable regions (V1-V7) of *tprK* captured directly from 14 primary syphilis clinical samples. (XLSX)

S4 Table The amino acid sequences within the seven variable regions (V1-V7) of *tprK* captured directly from 14 primary syphilis clinical samples. * indicates synonymous nucleotide sequences within the same strain.
(XLSX)
