# Supplementary figures and images for: Profile of the tprK gene in primary syphilis patients based on next-generation sequencing
Source: PLoS Negl Trop Dis. 2019 Feb 21;13(2):e0006855. doi: 10.1371/journal.pntd.0006855 (PMC6400401; doi:10.1371/journal.pntd.0006855)

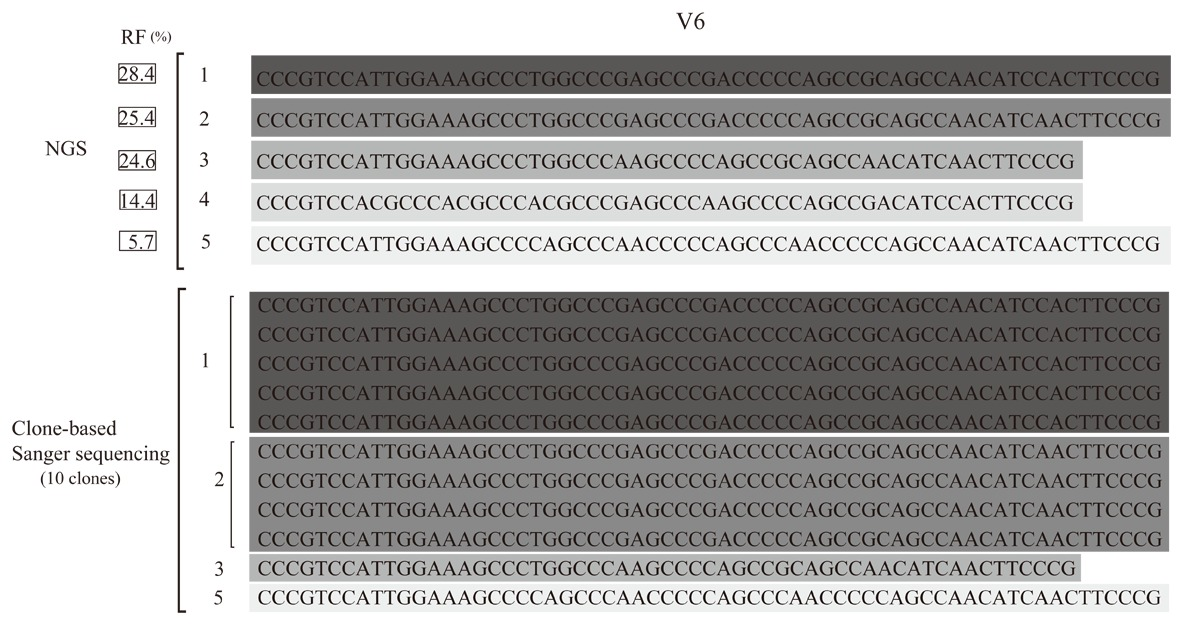

Supplement: S1 Fig — RF values indicate the relative frequency of each sequence. (TIF) [file pntd.0006855.s001.tif]
