# Supplementary material for: Profile of the tprK gene in primary syphilis patients based on next-generation sequencing
Source: PLoS Negl Trop Dis. 2019 Feb 21;13(2):e0006855. doi: 10.1371/journal.pntd.0006855 (PMC6400401; doi:10.1371/journal.pntd.0006855)
Supplement: S1 Table — (DOCX) [file pntd.0006855.s002.docx]

**S1 Table. The primers for *tprK* amplification**

| Primer | Purpose | Sequence (5’→3’) |
| --- | --- | --- |
| *tprK*-S | Amplification of *tprK* ORF | ACCGGGCATGAATTTTCTTT |
| *tprK*-As |  | CCATACATCCCTACCAAATCA |
| *tprK*-frag1-S | Amplification of *tprK* fragment1 | ATGATTGACCCATCTGCCAC |
| *tprK*-frag1-As |  | GTAGGCCCCATAACAGTGCA |
| *tprK*-frag2-S | Amplification of *tprK* fragment2 | GGTGGAGCAAAGTTTGACAC |
| *tprK*-frag2-As |  | TTAATGTATTCCTGCACGCC |
| *tprK*-frag3-S | Amplification of *tprK* fragment3 | GAAGATGGCGTGCAGGAATA |
| *tprK*-frag3-As |  | TCAACACCCAAATCAAGACC |
| *tprK*-frag4-S | Amplification of *tprK* fragment4 | TATTAAGCTCGAAACCAAGG |
| *tprK*-frag4-As |  | CCAAATCAAGCGACATGCCC |
